# Evaluation of the Diagnostic Value of Non-criteria Antibodies for Antiphospholipid Syndrome Patients in a Chinese Cohort

**DOI:** 10.3389/fimmu.2021.741369

**Published:** 2021-09-10

**Authors:** Chaojun Hu, Siting Li, Zhijuan Xie, Hanxiao You, Hui Jiang, Yu Shi, Wanting Qi, Jiuliang Zhao, Qian Wang, Xinping Tian, Mengtao Li, Yan Zhao, Xiaofeng Zeng

**Affiliations:** ^1^Department of Rheumatology, Peking Union Medical College Hospital, Peking Union Medical College and Chinese Academy of Medical Sciences; Key Laboratory of Rheumatology and Clinical Immunology, Ministry of Education, Beijing, China; ^2^National Clinical Research Center for Dermatologic and Immunologic Diseases, Beijing, China

**Keywords:** antiphospholipid syndrome, antiphospholipid antibodies, immunoglobulin A, anti-phosphatidylserine/prothrombin, anti-annexin V

## Abstract

**Objective:**

Although specific anti-phospholipid antibodies (aPLs) have been used in the diagnosis of the antiphospholipid syndrome (APS) for years, new biomarkers are required to increase its diagnostic and risk-predictive power. This study aimed to explore the value of several non-criteria aPLs in a Chinese cohort.

**Methods:**

A total of 312 subjects, namely, 100 patients diagnosed with primary APS, 51 with APS secondary to SLE, 71 with SLE, and 90 healthy controls, were recruited. Serum anticardiolipin (aCL) IgG/IgM/IgA, anti-β2-glycoprotein I (aβ2GPI) IgG/IgM/IgA, anti-phosphatidylserine/prothrombin antibodies (aPS/PT) IgG/IgM, and anti-annexin A5 antibodies (aAnxV) IgG/IgM were tested using ELISA kits.

**Results:**

Of the total number of patients, 30.46% and 6.62% with APS were positive for aCL or aβ2GPI IgA, respectively, while 39.07% and 24.50% were positive for aAnxV or aPS/PT for at least one antibody (IgG or IgM). The addition test of aCL IgA and aAnxV IgM assists in identifying seronegative APS patients, and IgG aPS/PT was linked to stroke.

**Conclusion:**

Detection of aCL IgA, aβ2GPI IgA, aAnxV IgG/M, and aPS/PT IgG/M as a biomarker provides additive value in APS diagnosis and would help in risk prediction for APS patients in medical practice.

## Introduction

The antiphospholipid syndrome (APS) is a systemic autoimmune disease characterized by thrombosis and/or pregnancy morbidity with the persistent presence of medium or high titer of antiphospholipid antibodies (aPLs). The 2006 APS classification criteria (Sydney criteria) have been widely accepted in APS diagnosis, where at least one of the clinical criteria, as well as one of laboratory criteria including lupus anticoagulant (LA), high level of anti-cardiolipin (aCL), and anti-β2 glycoprotein-I (aβ2GPI) immunoglobulin isotype G (IgG) or M (IgM), should be present (1). Despite its wide use in clinical practice, patients could remain persistently negative for criteria aPLs yet show typical APS clinical manifestations [defined as seronegative APS, SNAPS (2)], and a broader range of diagnostic biomarkers are required (3). Apart from standard criteria, other non-criteria clinical and laboratory features have been found associated with APS in numerous studies, such as heart valve disease, thrombocytopenia, neurological manifestations, anti-CL or anti-β2GpI IgA, anti-phosphatidylserine–prothrombin (aPS/PT) complex, and anti-annexin A5 antibodies (aAnxV) (4, 5). Besides APS diagnosis, evaluation of non-criteria aPLs could also contribute to prognosis and risk assessment for associated clinical manifestations (6).

More specifically, numerous studies have been conducted to investigate the diagnostic value of aCL/aβ2GpI IgA for APS, which received contradictory results (7). Nevertheless, testing of IgA had been recommended by guidelines when criterial aPLs remained negative (8). In addition, aAnxV and aPS/PT are receiving continuous attention in recent years. AnxV is a phospholipid-binding protein highly expressed in vascular endothelial cells. It could bind tightly to exposed anionic phospholipids and assemble into a shield, which may prevent phospholipid-dependent coagulation reaction (9, 10). In a systematic review, AnxV resistance has been observed and analyzed to have a higher prevalence in APS compared to disease controls (11) and has been reported to be linked to a hypercoagulable state as well as obstetric complications in APS patients (12, 13). Furthermore, its anticoagulant activity was reduced by plasmas of patients with APS and thromboembolism (14), and loss of maternal aAnxV increased the chance of placental platelet thrombosis and fetal loss (15). However, other studies found no significant association between thrombotic event or adverse pregnancy manifestations (16, 17).

Prothrombin is another phospholipid-binding protein that forms a complex and is often co-detected of antibodies together with phosphatidylserine (aPS/PT). An international multicenter study confirmed the contribution of aPS/PT IgG in APS diagnosis IgG (18). Concerning its relation with clinical features such as thrombotic events or obstetric complications, conflicting results had been shown and confirmation is still needed (19, 20). Nevertheless, numerous studies have indicated a strong correlation between aPS/PT and LA (21, 22). In addition, a higher level of aPS/PT was observed to be associated with high-risk “triple positive” patients (LA+, aCL IgG and/or IgM +, and aβ2GPI IgG and/or IgM+) (23), and may also add value to identification of SNAPS (3).

Study design, including detection method, patient stratification, population heterogeneity, and other factors, may lead to contradictory results in different studies. Regarding the Chinese population, a previous study indicated an increase of both IgG and IgM aAnxV in primary APS patients and APS associated with other diseases. Significant associations were also observed between IgG aANxV and thrombotic events (24). Additionally, assessment of the diagnostic performance of aPS/PT revealed a significant correlation between thrombotic events and pregnancy loss with IgG aPS/PT (25, 26), which was confirmed by a recent study (27). Concerning aCL/aβ2GpI IgA, a study recently conducted by us in a large Chinese population revealed little added diagnostic value (28). Few studies have explored all of the above non-criteria autoantibodies in the same patient groups, and their relations with more detailed clinical manifestations still need investigation. This study focused on evaluating the additive diagnostic value of aCL/aβ2GpI IgA, IgG and IgM for aANxV or aPS/PT to standard aPLs in a Chinese cohort. Correlation with clinical features including thrombotic events, obstetric complications, and microangiopathy was also explored.

## Patients and Methods

### Patients Recruitment

This was a single-center, prospective cohort study conducted at Peking Union Medical College Hospital (PUMCH) from May 2017 to January 2020. A total of 152 consecutive APS outpatient cases were included in this study, of which 100 patients had been diagnosed with primary APS (PAPS group) and 51 with APS secondary to SLE (SAPS group). A total of 71 SLE patients (SLE group) and 90 healthy controls (HC group) were also included and matched with APS groups for gender and age. Diagnosis of APS was defined by clinicians according to the 2006 Sydney revised classification criteria. Upon diagnosis, sera samples were collected at the outpatient clinic and immediately profiled of aPL antibodies at the Key Laboratory of Department of Rheumatology, Peking Union Medical College Hospital (PUMCH). Besides aPL serology, history of clinical manifestations was recorded for PAPS, SAPS, and SLE groups, including thrombosis (arterial or venous), pregnancy morbidity, microangiography (i.e., thrombocytopenia, autoimmune hemolytic anemia), and history of adverse pregnancy. For the HC group, only aPL serology information was present. The study was approved by the ethics committee at PUMCH and fulfilled the ethical guidelines of the declaration of Helsinki. All subjects gave written informed consent.

### Laboratory Tests

IgG, IgM, and IgA isotypes of aCL and aβ2GPI, IgG and IgM isotypes of aPS/PT and aAnxV were analyzed with AESKULISA^®^ ELISA Test Kits provided by Aesku. Diagnostics GmbH & Co. KG (Wendelsheim, Germany). Cutoff value was defined as 18 U/ml as recommended by the manufacturer. Lupus anticoagulant was detected and evaluated at the Key Laboratory according to the ISTH recommendations measuring Dilute Russell viper venom time (dRVVT)/activated partial thromboplastin time (>1.20 as positive) (29). Diagnosis of SLE was based on the 1997 ACR criteria and confirmed by the 2019 EULAR/ACR criteria.

### Statistical Analysis

Statistical analysis was performed using SPSS 26.0 or R (version 3.6.2). The *χ* ^2^ test or Fisher’s exact test was used for comparison of categorical variables, and Wilcoxon test was used for continuous variables after normality was explored with the Shapiro–Wilk test. Sensitivities, specificities, and accuracies in APS diagnosis were compared in the McNemar test. Youden Index, positive and negative predictive values (PPV and NPV), and odds ratio (OR) with 95% confidence interval (95% CI) were also shown. Receiver operating characteristic (ROC) curves of individual aPL as well as logistic regression analysis of aPLs profile were used to calculate the area under the curve (AUC), with 95% CI shown. Associations between aPL isotype positivity and clinical manifestation in patients with APS were explored and displayed in 95% CI. Two-tailed values of *p* less than 0.05 were considered statistically significant.

## Results

### Patient Characteristics

Among 151 APS patients, there were 63 (63.0%) females for PAPS, 45 (88.2%) for SAPS, and the mean age for each was 36.3 and 32.9 years (**Table 1**). The mean age was 30.1 years in the SLE group, of which 61 (85.9%) were female, while the HC group had 41 (45.6%) females and a mean age of 43.4. A significant difference of female:male ratio was observed between PAPS and SAPS (*χ* ^2^ = 10.560, *p* = 0.001). Clinical manifestations were recorded for both APS and SLE patients and were selectively shown. Thrombosis was most commonly present, with 80 (80.0%) for PAPS and 74.5% for SAPS, but not in the SLE group. Patients were recorded for history of arterial or venous thrombotic events, pregnancy morbidity, microangiopathy, history of adverse pregnancy, and LA. Of all the clinical manifestations, prevalence of adverse pregnancy history was significantly different between the PAPS and SAPS group (*χ* ^2^ = 3.922, *p* = 0.048).

**Table 1:** Demographic and clinical variables of subjects (*n* = 312).

	APS (151)		SLE (71)	Healthy controls (90)
	Primary (100)	Secondary (51)		
Gender (female/male)	63/37	45/6	61/10	41/49
Mean age (years ± SD)	36.3 ± 12.1	32.9 ± 10.2	30.1 ± 8.2	43.4 ± 12.2
**Clinical manifestations**				
Thrombosis, *n* (%)	80 (80.0%)	38 (74.5%)	0	NA
Pregnancy morbidity, *n* (%)	33 (52.4%)	16 (35.6%)	0	NA
Thrombosis + pregnancy morbidity, *n* (%)	13 (20.6%)	3 (6.7%)	0	NA
LA, *n* (%)	73 (73.0%)	44 (86.3%)	17 (23.9%)	NA
History of arterial thrombosis, *n* (%)	43 (43.0%)	21 (41.2%)	0	NA
Stroke, *n* (%)	15 (15.0%)	4 (7.8%)	0	NA
Coronary heart disease, *n* (%)	5 (5.0%)	0	0	NA
Eye involvement, *n* (%)	3 (3.0%)	1 (2.0%)		
Lower limb artery occlusion, *n* (%)	1 (1.0%)	0	0	NA
History of venous thrombosis, *n* (%)	47 (47.0%)	24 (47.1%)	0	NA
Deep vein thrombosis, *n* (%)	19 (19.0%)	7 (13.7%)	0	NA
Pulmonary embolism, *n* (%)	19 (19.0%)	2 (3.9%)	0	NA
Upper limb vein thrombosis, *n* (%)	0	1 (2.0%)	0	NA
Renal vein thrombosis, *n* (%)	1 (1.0%)	0	0	NA
Portal vein thrombosis, *n* (%)	4 (4.0%)	1 (2.0%)	0	NA
Cerebral venous and sinus thrombosis, *n* (%)	3 (3.0%)	1 (2.0%)	0	NA
Central retinal venous occlusion, *n* (%)	1 (1.0%)	0	0	NA
Microangiopathy, *n* (%)	11 (11.0%)	13 (25.5%)	0	NA
Non-stroke CNS manifestations, *n* (%)	4 (4.0%)	4 (7.8%)	0	NA
Heart valve disease, *n* (%)	0	6 (11.8%)	0	NA
Antiphospholipid syndrome nephropathy, *n* (%)	6 (6.0%)	2 (3.8%)	0	NA
Hemolytic uremic syndrome, *n* (%)	1 (1.0%)	0	0	NA
Thrombotic microangiopathy, *n* (%)	0	1 (2.0%)	0	NA
Hematological disorder, *n* (%)	39 (39.0%)	33 (64.7%)	0	NA
Thrombocytopenia, *n* (%)	38 (38%)	*28 (54.9%)	21 (29.6%)	NA
Autoimmune hemolytic anemia, *n* (%)	1 (1.0%)	5 (9.8%)	0	NA
History of adverse pregnancy, *n* (%)	37 (58.7%)	20 (44.4%)	4 (5.6%)	NA
Early fetal loss (<10 weeks), *n* (%)	12 (19.0%)	8 (17.8%)	4 (5.6%)	NA
Late fetal loss (10–28 weeks), *n* (%)	19 (30.2%)	12 (26.7%)	0	NA
Recurrent fetal loss (>1 time), *n* (%)	11 (17.5%)	5 (11.1%)	2 (3.3%)	NA
Placental insufficiency, *n* (%)	14 (22.2%)	7 (15.6%)	0	NA

*p = 0.048, significantly different from primary APS; NA, not available.

### Predictive Power of aPLs in APS Diagnosis

The diagnostic power of aPLs positivity (>18 U/ml) was evaluated for sensitivity, specificity, accuracy, Youden Index, PPV, NPV, and ORs in APS diagnosis from HC group in **Table 2**. For IgA, the sensitivity and accuracy of the combination of aCL IgG, IgM, or IgA were significantly higher than that of aCL IgG or IgM (*p* < 0.001), while specificity was lower (*p* = 0.031). A similar result was observed for aCL or aB2GpI IgG or IgM or IgA compared to aCL or aB2GpI IgG or IgM. As for aAnxV, the sensitivity and accuracy of aAnxV IgG or IgM were significantly higher than that of aB2GpI IgG or IgM (*p* < 0.001). In addition, a combination of aCL, aβ2GpI, or aAnxV IgG or IgM had significantly higher sensitivity (*p* = 0.016) compared to that of aCL or aβ2GpI IgG or IgM.

**Table 2:** The predictive value of different aPLs in APS diagnosis.

	Sensitivity (%)	Specificity (%)	Accuracy(%)	Youden Index	PPV (%)	NPV (%)	OR (95% CI)
aCL IgG	37.09	100.00	60.58	0.371	100.00	48.65	∞
aCL IgM	8.61	97.78	41.90	0.064	86.67	38.94	4.15 (0.91–18.81)
aCL IgG or IgM	41.06	97.78	62.24	0.389	96.88	49.72	30.65 (7.27–129.20)
aβ2GpI IgG	23.18	100.00	51.86	0.232	100.00	43.69	∞
aβ2GpI IgM	7.95	98.89	41.91	0.068	92.31	39.04	7.68 (0.98–60.12)
aβ2GpI IgG or IgM	29.14	98.89	55.19	0.28	97.78	45.41	36.60 (4.94–270.96)
aCL or aB2GpI IgG or IgM	43.05	97.78	63.48	0.408	97.01	50.57	33.26 (7.89–140.10)
aCL IgA	30.46	92.22	53.53	1.2268	86.79	44.15	5.19 (2.23–12.10)
aβ2GpI IgA	6.62	98.89	41.08	1.0551	90.91	38.70	6.31 (0.79–50.16)
aCL IgG or IgM or IgA	51.66	91.11	66.39	1.4277	90.70	52.90	10.95 (4.96–24.21)
aβ2GpI IgG or IgM or IgA	31.79	97.78	56.43	1.2957	96.00	46.07	20.51 (4.85–86.79)
aCL or aB2GpI IgG or IgM or IgA	53.64	91.11	67.63		91.01	53.95	11.86 (5.37–26.22)
P_1_	**<0.001**	**0.031**	0.052				
P_2_	0.125	1.000	0.375				
P_3_	**<0.001**	**0.031**	0.052				
aPS/PT IgG	18.54	96.67	47.72	0.152	90.32	41.43	6.60 (1.95–22.40)
aPS/PT IgM	7.28	98.89	41.49	0.062	91.67	38.86	6.99 (0.89–55.10)
aPS/PT IgG or IgM	24.50	95.56	51.03	0.201	90.24	43.00	6.99 (2.40–20.32)
aCL, aB2GpI, or aPS/PT IgG or IgM	45.70	94.44	63.90	0.401	93.24	50.90	14.31 (5.49–37.25)
P_1_’	**<0.001**	0.625	**<0.001**				
P_2_’	0.167	0.375	0.064				
P_3_’	0.125	0.250	1.000				
aAnxV IgG	30.46	100.00	56.43	0.305	100.00	46.15	∞
aAnxV IgM	16.56	96.67	46.47	0.133	89.29	40.85	5.75 (1.69–19.70)
aAnxV IgG or IgM	39.07	96.67	60.58	0.358	95.16	48.60	18.60 (5.62–61.53)
aCL, aβ2GpI, or aAnxV IgG or IgM	47.68	96.67	65.98	0.444	96.00	52.41	26.43 (8.01–87.26)
P_1_’’	0.648	1.000	0.503				
P_2_’’	**<0.001**	0.500	**0.007**				
P_3_’’	**0.016**	1.000	0.070				

PPV, positive predictive value; NPV, negative predictive value; OR, odds ratio; CI, confidence interval. p-values of sensitivity, specificity, and accuracy are calculated with McNemar test.

P_1_: Comparison of result of aCL IgG or IgM or IgA to aCL IgG or IgM; P_2_: Comparison of result of aβ2GpI IgG or IgM or IgA to aβ2GpI IgG or IgM; P_3_: Comparison of result of aCL or aβ2GpI IgG or IgM or IgA to aCL or aβ2GpI IgG or IgM; P_1_’: Comparison of result of aPS/PT IgG or IgM to aCL IgG or IgM; P_2_’: Comparison of result of aPS/PT IgG or IgM to aβ2GpI IgG or IgM; P_3_’: Comparison of result of aCL, aβ2GpI, or aPS/PT IgG or IgM to aCL or aβ2GpI IgG or IgM; P_1_’’: Comparison of result of aAnxV IgG or IgM to aCL IgG or IgM; P_2_’’: Comparison of result of aAnxV IgG or IgM to aβ2GpI IgG or IgM; P_3_’’: Comparison of result of aCL, aB2GpI, or aAnxV IgG or IgM to aCL or aβ2GpI IgG or IgM. Odds ratios (ORs) with 95% confidence intervals (CIs) are shown.

Bold values mean P < 0.05.

**Figure 1:**
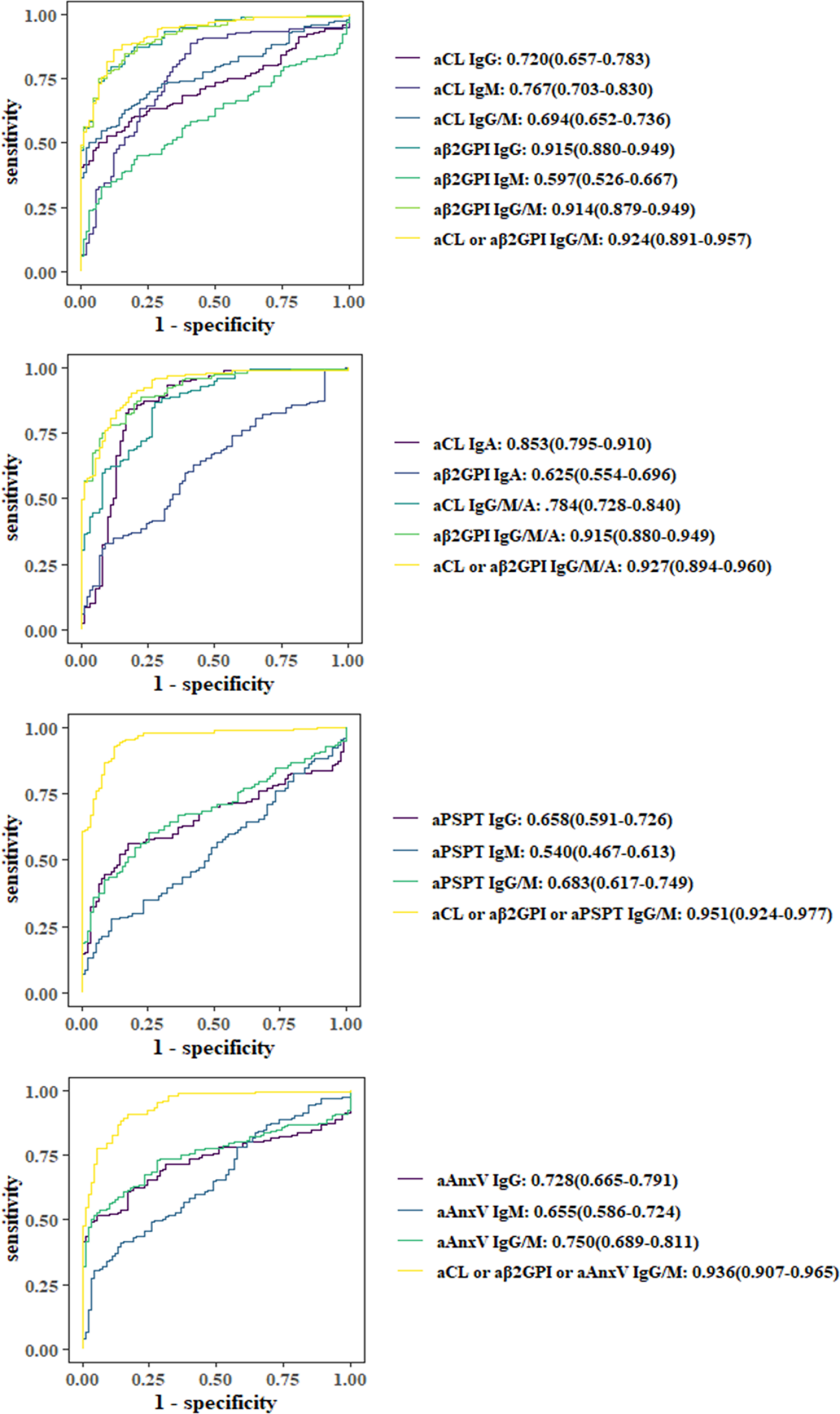
Comparison of receiver operating characteristic (ROC) curves and area under the curve (AUC). ORs with 95% CIs are shown.

As illustrated in **Figure 1**, ROC curves were applied to evaluate the predictive value of aPLs or their combined positivity. Among individual aPLs, aβ2GP1 IgG (0.915), aCL IgA (0.853), aCL IgM (0.767), and aAnxV IgG (0.728) had the largest AUC values. Adding IgA, aAnxV or aPS/PT IgG or IgM to aCL or aβ2GpI IgG or IgM would both increase AUC (0.927, 0.951, and 0.936, compared to 0.925).

### Cross-Positivity Analysis for Four aPLs in APS Patients

**Figure 2:**
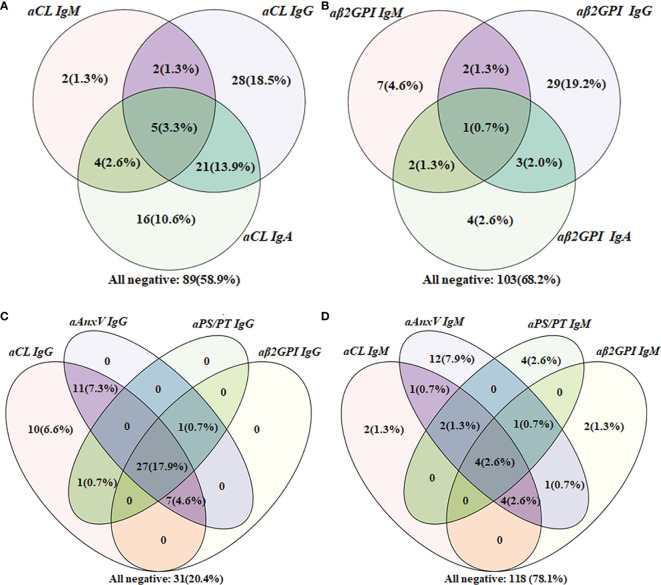
Venn diagram of aPLs cross-positivity analysis in the APS group (*n* = 151). **(A)** Cross-positivity for aCL; **(B)** cross-positivity for aβ2GpI; **(C)** cross-positivity for IgG; **(D)** cross-positivity for IgM.

Among 151 APS patients, cross-positivity of IgG, IgM, or IgA for aCL or aβ2GpI (a and b), as well as IgG or IgM for each of the four aPLs (c and d) were demonstrated with the Venn diagram in **Figure 2**. For patients positive for aCL, 16 were positive only for IgA. Concerning IgG isotype, aCL and aAnxV IgG were most often positive among APS patients. As for the IgM isotype, there were 12 (7.9%) patients who tested positive only for aAnxV, and 4 (2.6%) were positive only for aPS/PT.

For APS patients, 43 (28.5%) were positive for more than one non-criteria aPLs. Besides, the number of patients positive only for one of the five non-criteria aPLs was also calculated. A total of 11 patients positive only for aCL IgA, 9 for aAnxV IgG, 5 for aAnxV IgM, 3 for aPS/PT IgM, 1 for aPS/PT IgG, and 1 for aβ2GpI IgA were observed among these patients.

### Distribution of Antiphospholipid Antibodies

**Figure 3:**
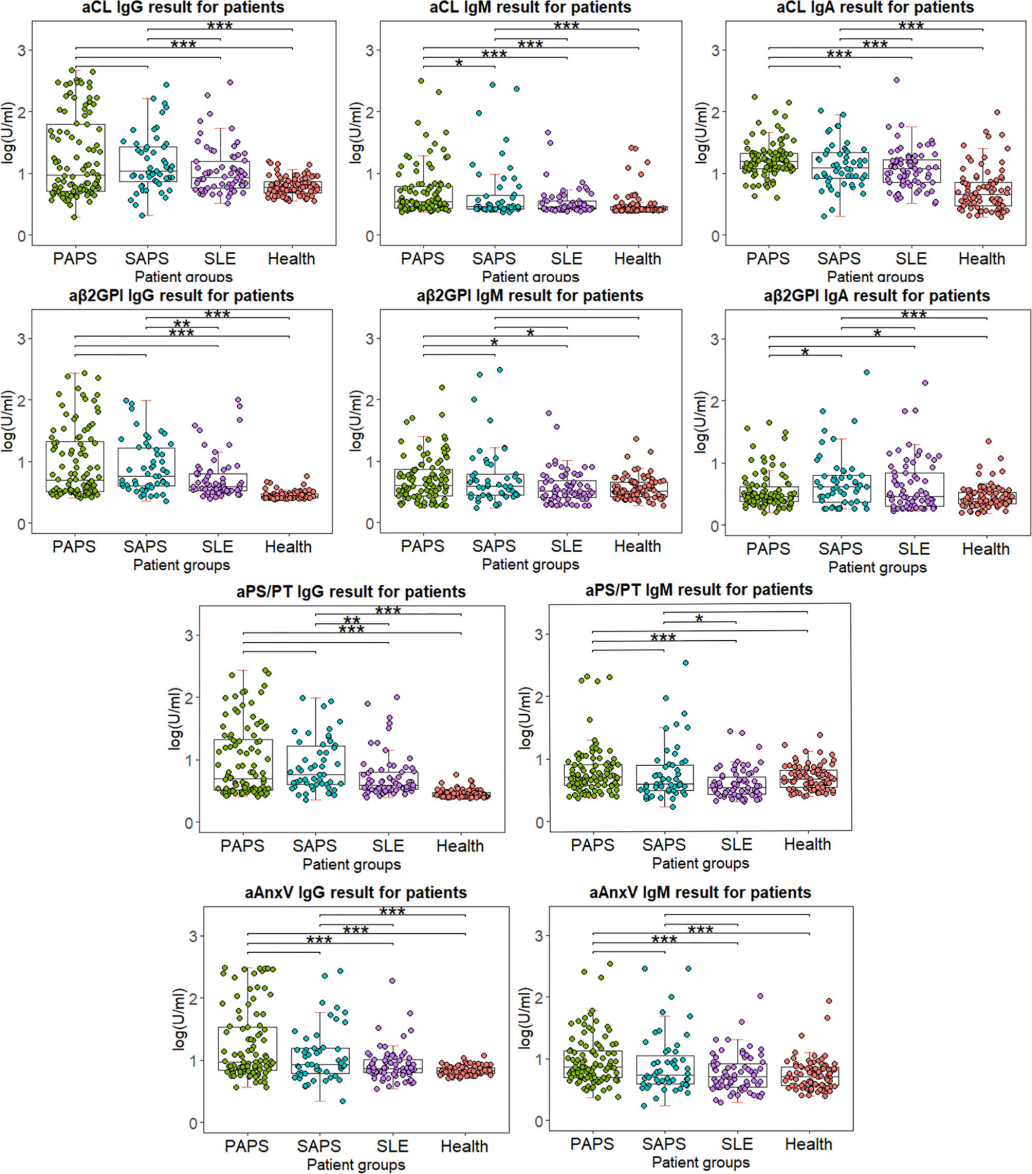
Distribution of IgG and IgM for four antibodies among different patient groups. Test results are calculated using lg(test value + 2), in order for the value to be shown in positive number. Wilcox’s test is conducted comparing primary or secondary APS results to other patient groups. *p < 0.05, **p < 0.01, ***p < 0.001.

The distribution of all criterial or non-criteria aPLs among different patient groups is shown in **Figure 3**. Levels of aPLs were calculated with (log(test value + 2)U/ml). The results of primary or secondary APS were compared to other groups. No significant difference was observed between primary and secondary APS, except for higher aCL IgM for PAPS (*p* = 0.029) and aβ2GpI IgA for SAPS (*p* = 0.043). Compared to HC, levels of IgG and IgA were significantly higher for four aPLs in both PAPS and SAPS group. However, IgM results varied for different aPLs.

### Clinical Manifestations of Different aPLs in APS Patients

Correlations between different aPLs and LA or clinical manifestations are shown with odds ratios in **Table 3**. Presence of LA was significantly associated with IgG of aCL (ORs 9.0, 95% CI 2.6–31.0), aβ2GPI (ORs 14.1, 95% CI 1.9–107.2), aPS/PT (ORs 4.7, 95% CI 1.1–21.2), and aAnxV (ORs 21.5, 95% CI 2.8–163.0). Among all vascular events, stroke was significantly associated with aβ2GPI IgG (ORs 2.8, 95% CI 1.0–7.7) as well as aPS/PT IgG (ORs 3.1, 95% CI 1.1–8.7). Additionally, aPS/PT IgM was reversely associated with pregnancy loss in women (ORs 0.6, 95% CI 0.5–0.7). LA positive was significantly related to thrombotic events (ORs 4.0, 95% CI 1.7–9.5), TP (ORs 4.1, 95% CI 1.7–10.2), and stroke (ORs 1.7, 95% CI 1.5–6.2).

**Table 3:** Correlations between different aPLs and clinical manifestations among APS patients (*n* = 151).

	Thrombosis	Arterial thrombosis	Venous thrombosis		Pregnancy morbidity	Pregnancy loss	TP	Microangiopathy	Stroke	LA
aCL IgG	1.3 (0.6–3.1)	1.6 (0.8–3.2)	0.8 (0.4–1.6)	1.2 (0.6–2.7)		1.0 (0.5–2.3)	1.3 (0.7–2.6)	1.3 (0.6–2.4)	2.1 (0.8–5.5)	9.0* (2.6–31.0)
aCL IgM	0.6 (0.2–1.9)	0.8 (0.3–2.7)	0.7 (0.2–2.3)	1.0 (0.3–3.5)		0.5 (0.1–2.1)	1.6 (0.5–4.9)	0.5 (0.2–1.6)	1.3 (0.3–6.3)	3.9 (0.5–31.3)
aCL IgA	0.6 (0.2–1.2)	0.8 (0.4–1.5)	0.8 (0.4–1.5)	0.9 (0.4–1.9)		1.5 (0.7–3.2)	0.8 (0.4–1.5)	0.6 (0.3–1.2)	1.8 (0.7–4.8)	1.2 (0.5–2.5)
aβ2GPI IgG	1.7 (0.6–5.0)	1.6 (0.8–3.4)	1.0 (0.5–2.1)	1.1 (0.5–2.9)		0.9 (0.4–2.4)	1.3 (0.6–2.8)	1.4 (0.6–3.0)	2.8* (1.0–7.7)	14.1* (1.9–107.2)
aβ2GPI IgM	1.3 (0.3–6.5)	0.4 (0.1–1.6)	2.5 (0.7–8.6)	0.9 (0.2–4.2)		0.6 (0.1–3.0)	1.9 (0.6–6.3)	1.2 (0.4–4.1)	2.5 (0.6–10.5)	1.5 (0.3–7.5)
aβ2GPI IgA	1.1 (0.2–5.2)	0.9 (0.2–3.3)	1.8 (0.5–6.7)	1.0 (1.0–1.1)		0.7 (0.2–3.0)	0.8 (0.2–3.1)	0.6 (0.2–1.0)	0.8 (0.1–6.4)	2.9 (0.4–23.4)
aPS/PT IgG	1.3 (0.4–3.6)	1.7 (0.8–4.0)	0.7 (0.3–1.6)	1.3 (0.5–3.4)		0.9 (0.3–2.6)	1.1 (0.5–2.6)	1.4 (0.6–3.3)	3.1* (1.1–8.7)	4.7* (1.1–21.2)
aPS/PT IgM	1.2 (0.2–5.8)	0.8 (0.2–2.7)	2.1 (0.6–7.6)	0.4 (0.1–2.0)		0.6* (0.5–0.7)	1.6 (0.5–5.5)	0.3 (0.1–1.2)	0.7 (0.1–5.6)	3.2 (0.4–26.0)
aAnxV IgG	1.7 (0.7–4.3)	1.8 (0.9–3.6)	0.8 (0.4–1.7)	1.0 (0.4–2.3)		0.7 (0.3–1.7)	1.9 (0.9–3.7)	1.5 (0.8–3.1)	2.3 (0.9–6.1)	21.5* (2.8–163.0)
aAnxV IgM	1.1 (0.4–3.1)	1.3 (0.6–3.1)	0.7 (0.3–1.8)	1.0 (0.3–2.6)		0.9 (0.3–2.6)	1.8 (0.8–4.3)	1.4 (0.6–3.3)	2.7 (0.9–8.1)	2.5 (0.7–8.9)
LA	4.0* (1.7–9.5)	1.3 (0.6–2.9)	2.7* (1.2–6.1)	0.5 (0.2–1.1)		0.9 (0.4–2.2)	4.1* (1.7–10.2)	1.5 (0.7–3.2)	1.7* (1.5–6.2)	–

Odds ratios (ORs) with 95% confidence intervals (CIs) are shown. *p < 0.05.

## Discussion

APS is an autoimmune disease featuring thrombosis and/or pregnancy morbidity, which may lead to severe consequences. Detection of aCL and aβ2GPI as the golden standard in APS diagnosis is not satisfactory in the clinical scenario, and various potential aPLs have been extensively explored.

In this study, the diagnostic value of IgA for aCL or aβ2GPI and of IgG/IgM for aANxV or aPS/PT was evaluated in APS patients. In brief, 45.70% and 6.62% of patients with APS were positive for aCL or aβ2GPI IgA, respectively, while 30.46% and 24.50% were positive for aAnxV or aPS/PT for at least one antibody (IgG or IgM). Adding IgA to criterial aPLs could increase the sensitivity in APS diagnosis. Detection of aANxV or aPS/PT, especially aAnxV IgG, could add value to diagnosis. IgG of aANxV or aPS/PT was significantly associated with LA, and IgG aANxV was linked to stroke.

Analysis of the predictive power indicates that although aCL IgA had relatively low specificity, adding IgA to aCL IgG or IgM/aCL or aβ2GpI IgG or IgM test could increase test sensitivity (*p* < 0.001). The sensitivity (39.07% compared to 29.14%, *p* < 0.001) and accuracy (60.58% compared to 55.19%, *p* = 0.007) of aAnxV IgG or IgM were both significantly higher than that of aβ2GPI IgG or IgM. Moreover, combination of aCL, aβ2GpI, or aAnxV IgG or IgM had significantly higher sensitivity (47.7% compared to 43.0%, *p* = 0.016) than that of aCL or aβ2GpI IgG or IgM. Statistical results suggested that adding aAnxV IgG or IgM to aCL or aβ2GpI IgG or IgM would both increase diagnostic value besides criterial antibodies. Meanwhile, there was no significant decrease in specificity (96.67%).

The result was further illustrated with ROC curves for each aPL and their combination. AUC of aCL IgA and aAnxV IgG ranked second and third (0.853 and 0.728) among individual aPLs. Statistical analysis showed that the addition of IgA to aCL IgG/IgM would significantly increase diagnostic power (AUC value 0.784 compared to 0.694). The addition of aAnxV or aPS/PT to aCL/aβ2GpI IgG/IgM would also significantly increase diagnostic power (AUC value 0.951 compared to 0.924).

The Venn diagram indicated the additive value of new aPLs from another perspective. Positive only for IgA isotype could point out an extra number of patients for both aCL (16, 10.6%) and aβ2GPI (4, 2.6%). Additionally, the number of patients positive for aAnxV IgG, aAnxV IgM, and aPS/PT IgM outperformed those of aβ2GPI, indicating their importance in APS clinical diagnosis. The result suggested that additional tests for non-criteria aPLs may provide a unique value in the identification of SNAPS patients.

Besides predictive power, distribution, and comparison of aPLs among different patient groups were also examined. Between PAPS and SAPS, little significant difference was observed except for aCL IgM (*p* = 0.029) and aβ2GpI IgA (*p* = 0.043). Between PAPS and SLE, significantly higher titer of IgM aCL, IgA aCL, IgM aPS/PT, IgG AnxV, and IgM AnxV was observed (*p* < 0.001). As for SAPS and SLE, only IgM aPS/PT showed a significant difference (*p* = 0.015). The results implied that both criterial and non-criteria aPLs had difficulty in distinguishing APS from SLE or APS secondary to SLE. Indeed, baseline information suggested little difference between PAPS and SAPS patients in age and most clinical manifestations (**Table 1**). It had been estimated in previous studies that around 40% of patients with SLE have aPL, and APS may develop in up to 50%–70% of patients with both SLE and aPL (30). As all SLE patients were matched with APS groups for gender and age, we believed that this similarity in aPL results reflected the unique characteristic of aPL distribution in our population. Nevertheless, levels of IgG for four aPLs were significantly higher in both PAPS and SAPS group compared to HC, which suggested their diagnostic value.

Finally, the relationship between aPLs and related clinical manifestations was calculated. In this study, no significant association was found between aPLs with any thrombotic events, which was contradictory with results from some previous studies conducted in the Chinese population (24–26). Consistent with most previous findings, LA was strongly associated with thrombotic events including stroke (31). As the strongest predictor of APS-related features, LA was also a strong indicator of TP. Concerning obstetric complication, aPS/PT IgM was reversely associated with pregnancy loss in women (ORs 0.6, 95% CI 0.5–0.7), which also showed conflicting results (25, 32, 33). For aAnxV, similar to a previous study, no significant relationship was observed (24). The different results might be due to the detection system. ELISA was chosen in this study, and the cutoff value provided by the manufacturer (18 U/ml for all the aPLs) may not reflect real aPL distribution in local population. As illustrated in **Figure 2**, 31 patients were negative for all IgG, while as many as 118 patients were negative for all IgM. The cutoff value provided by the manufacturer might be overly strict and impact test sensitivity. It could be more suitable if the 99th percentile strategy was adopted first to identify cutoff points for each aPL. Regarding vascular manifestation, a significant relationship with aPLs (aβ2GPI IgG and aPS/PT IgG) was present for stroke. A previous review had estimated an aPL positivity of 17% in patients with juvenile stroke (<50 years of age) (34), and incidence of stroke was up to 20% among APS patients in another cohort (35). Although detection of aPS/PT alone may have less diagnostic value, it would still be valuable in risk prediction for and prevention of adverse clinical events. Additionally, the relationship between aAnxV and aPS/PT IgG and LA was confirmed in our studies, and LA was found to be associated with IgG of all four aPLs.

This study has some limitations. Compared to similar studies, the sensitivity for some autoantibodies is not very high, which may influence the results of sequence comparison. Since different detection methods and manufacturers vary greatly in antibody measurement, contradictory results could arise (36). In the future, quantitative/semi-quantitative detection methods such as chemiluminescence analysis (CLIA) could be applied to reduce systemic detection error. In addition, both patients and healthy individuals involved in the study were relatively homogenous and may not reflect real-life condition. A larger sample size and inclusion of patients with a wider range of associated diseases or clinical features could further complement the study. Last but not least, the diagnostic power of other non-criteria aPLs such as aβ2GPI anti-domain I could also be explored with a similar procedure.

## Conclusion

In conclusion, detection of aCL IgA, aβ2GPI IgA, aAnxV IgG/M, and aPS/PT IgG/M as a biomarker provides additive value in APS diagnosis, especially aCL IgA and aAnxV IgG. Detecting aCL IgA and aAnxV IgM assists in identifying seronegative APS patients. IgG of aANxV or aPS/PT was significantly associated with LA, and IgG aANxV was linked to stroke, which would assist in risk prediction for APS patients in medical practice.

## Data Availability Statement

The raw data supporting the conclusions of this article will be made available by the authors, without undue reservation.

## Ethics Statement

The studies involving human participants were reviewed and approved by the Ethics Committee of Peking Union Medical College Hospital. The patients/participants provided their written informed consent to participate in this study.

## Author Contributions

All authors were involved in the design of this study. CH, SL, ZX, HY, HJ, and JZ contributed to the collection of blood samples and other experimental procedures. YS and WQ were involved in data collection and pre-processing. CH and SL analyzed the data and wrote the manuscript. JZ, QW, XT, ML, and YZ contributed to the recruitment of patients and evaluation of clinical data. All authors contributed to the article and approved the submitted version.

## Funding

This study was supported by the National Key Research and Development Program of China (2019YFC0840603, 2017YFC0907601, and 2017YFC0907602), the National Natural Science Foundation of China (81771780), and the CAMS Initiative for Innovative Medicine (2017-I2M-3-001 and 2019-I2M-2-008).

## Conflict of Interest

The authors declare that the research was conducted in the absence of any commercial or financial relationships that could be construed as a potential conflict of interest.

## Publisher’s Note

All claims expressed in this article are solely those of the authors and do not necessarily represent those of their affiliated organizations, or those of the publisher, the editors and the reviewers. Any product that may be evaluated in this article, or claim that may be made by its manufacturer, is not guaranteed or endorsed by the publisher.
